# Operationalisation of health equity principles in physiotherapy hospital triage policies

**DOI:** 10.1186/s12939-024-02249-6

**Published:** 2024-08-21

**Authors:** Lisa Pagano, Nick Glenn, Karen Hutchinson, Janet C. Long, Jeffrey Braithwaite, Mitchell N. Sarkies

**Affiliations:** 1 Australian Institute of Health Innovation, Faculty of Medicine, Health and Human Sciences, Macquarie University, Sydney, Australia; 2 Sydney School of Health Sciences, Faculty of Medicine and Health, University of Sydney, Sydney, Australia; 3 Central Coast Local Health District, Gosford, NSW Australia; 4 Central Coast Research Institute for Integrated Care, Gosford, NSW Australia; 5 Implementation Science Academy, Sydney Health Partners, University of Sydney, Sydney, Australia

**Keywords:** Equality, Prioritisation, Health Equity, Allied health, Resource allocation

## Abstract

**Background:**

Healthcare triage policies are vital for allocating limited resources fairly and equitably. Despite extensive studies of healthcare equity, consensus on the applied definition of equity in triage remains elusive. This study aimed to investigate how the principles of equity are operationalised in Australian hospital physiotherapy triage tools to guide resource distribution.

**Methods:**

A retrospective, qualitative content analysis of 13 triage policies from 10 hospitals across Australia was conducted. Triage policies from both inpatient and outpatient settings were sourced. Data were coded deductively using the five discrete domains of the multi-faceted operational definition of health equity posited by Lane et al. (2017): 1) point of equalisation in the health service supply/access/outcome chain, 2) need or potential to benefit, 3) groupings of equalisation, 4) caveats to equalisation, 5) close enough is good enough. Descriptive summative statistics were used to analyse and present the frequency of reported equity domains.

**Results:**

Within the included triage tools, four out of five domains of equity were evident in the included documents to guide decision making. Allocation based on perceived patient need and overall health outcomes were the central guiding principles across both inpatient and outpatient settings. Equal provision of service relative to patient need and reducing wait times were also prioritised. However, explicit inclusion of certain equity domains such as discrimination, ensuring equal capability to be healthy and other patient factors was limited.

**Conclusions:**

Physiotherapy triage policies consider various domains of equity to guide resource allocation decisions. Policymakers and service providers can use the insights gained from this study to review the application and operationalisation of equity principles within their healthcare systems through mechanisms such as patient triage tools.

**Supplementary Information:**

The online version contains supplementary material available at 10.1186/s12939-024-02249-6.

## Background

Triaging service provision is ubiquitous throughout the health system. Triage is commonly associated with prioritising the urgency of care in emergency departments; however, patients may enter a system of triage at any point of their care episode [1]. In critical care instances, triage tools can assist clinician decision making and ensure timely service provision to patients with the most urgent needs [1]. Triage may also be used to determine patient suitability for service referral [2] or the intensity of services required [3]. Given that the demand for healthcare services often exceeds their availability and how this demand is expected to grow due to chronic disease and an ageing population, increased attention has been directed towards the use of triage tools to allocate limited resources and prioritise patient care. Considering the widespread use of triage tools, it is important to ensure that they are designed to achieve the desired allocation of resources according to societal expectations and considerations of health equity.

Health equity is a multidimensional concept that aims to disrupt unequal treatment of healthcare consumers on a moral basis to ensure a state in which every individual has a fair opportunity to attain their highest level of health, regardless of socio-economic, demographic and geographic factors [4]. The application of health equity principles in triage policies and guidelines has been critically examined, especially in recent times in the context of COVID-19, where the availability of critical care resources in some countries was exceeded by demand, leading to the rationing of medical resources [5–7]. Triage tools may distribute scarce healthcare resources according to different principles, depending on the design or purpose of the tool. For example, triage tools that promote the reduction of healthcare gaps between groups would follow egalitarian principles whereas those tools that target the allocation of resources to those most likely to benefit, maximise the number of lives saved and minimise the dispersion of ill-health across the board would be grounded in utilitarian assumptions [8–10].

It has been argued that existing triage protocols may amplify health disparities and concerns have been raised regarding the use of different domains of health equity in these triage policies [5, 7]. For example, allocating resources with the assumption to ‘save the most lives’ may amplify disparities for disadvantaged groups [11]. By comparison, using random allocation of resources may result in fewer lives saved. The balance between promoting optimal health outcomes overall and promoting social justice can be difficult to determine, given the multitude of domains that could be considered when forming an operational definition of equity for health policy or service delivery. Lane and colleagues (2017) argue that health care policy makers should identify a single point of equalisation in service availability, delivery, use, or outcomes to guide service implementation decisions that seek to achieve health equity in a particular health service [9].

Allied health encompasses a diverse group of health professionals, such as physiotherapists (physical therapists) and occupational therapists, who provide a broad range of healthcare services across different settings [12, 13]. Allied health represents more than a quarter of the healthcare workforce and delivers over 3 million inpatient service events per year in both Australian acute and subacute settings [14]. Allied health triage tools detail a set of guidelines or criteria devised to determine the allocation of these resources and services across both inpatient and outpatient contexts. Tools can be arranged into various formats including single page risk matrices or multi-page guidelines. Clinicians may access these tools, typically stored in accessible databases or intranet systems within healthcare institutions, whenever they encounter patient referrals to help guide their clinical decision-making. For example, allied health practitioners rely on triage tools to prioritise service delivery to patients on weekends when there are less staff available. Since the allocation of many of these services are dictated by triage tools, it is important to consider how health equity has been operationalised in those tools. Previous studies have shown that allied health clinicians do consider equity principles when making resource allocating decisions, however, they are often reluctant to nominate one domain that should be the chief consideration when making these decisions [10]. This study aimed to identify the different domains of equity that have been operationalised in Australian hospital physiotherapy triage tools.

## Methods

### Study design and cohort selection

A retrospective, cross-sectional qualitative content analysis of official hospital policy tools referring to the physiotherapy triage process was conducted [15]. A purposive convenience sample of tools for inpatient or outpatient physiotherapy services were obtained from departments in rural and metropolitan hospitals across Australia. Either public or private hospitals listed on the Australian Institute of Health and Welfare MyHospitals registry were considered eligible [16]. A range of triage policy tools were sought to be representative of allied health services from different local hospital networks within the Australian healthcare system. The research team (MS, NG, KH) contacted physiotherapy departments from existing professional networks via email from 26th October 2021 to 1st December 2021 to obtain official policy tools referring to the triage process. Initially, 17 departments were contacted, and a snowballing approach was then used where those contacted were encouraged to contact their networks to expand the potential reach of the tools collected. Documents were stored in a secure location only accessible to the research team and all institutions remained de-identified.

### Data analysis

Data analysis followed a deductive thematic coding procedure [17] using the 21 discrete domains of the multi-faceted operational definition of equity posited by Lane et al. [9] as the coding scheme (Table 1) [9]. Tools were read multiple times prior to coding to ensure familiarity with the data. Line-by-line coding was then conducted where implicit or explicit definitions of equity within each tool were compared to the coding scheme and assigned a relevant code from one of the 21 established domains of equity [9]. Verbatim definitions used in the tools were reported in contrast to the coded definition to enhance methodological transparency. Following coding, the assigned codes were organised into categories reflecting the 21 established domains, grouping similar themes for a structured analysis of the data.

**Table 1:** The domains of equity and sub-themes of equity

Five domains of equity	Sub-themes of equity
**Domain 1: Point of equalisation in the health service supply/access/outcome chain** *The points within the healthcare system where resources are distributed in a manner that aims to reduce disparities and achieve a level playing field, culminating in the health states attained by people using these services*	Opportunity to access services *(individuals have equal opportunity to access care)*
	Burden in accessing services *(equality in the burden individuals’ face e.g. cost to access care)*
	Quantity for equivalent need *(equal amount of service available to groups or individuals)*
	Use of service
	Quality of service received
	Access regardless of capacity to pay
	Duration of wait for service
	Cost burden *(cost burdens to making a service available should be equalised)*
	Cross subsidy
	Cost effectiveness
	Health outcome *(obtaining equal health outcomes for individuals or equal improvement in an individuals’ health state)*
	Reach *(number of people to benefit)*
**Domain 2: Need or potential to benefit** *Priorisation of individuals based on their need or potential to benefit from care or resources received*	Perceived health need *(reduction in an individuals’ health state where intervention may improve outcomes)*
	Greater potential to benefit *(prioritise individuals based on the amount of benefit likely to be received from care)*
	Contribution to society *(care provided to individuals that have potential to contribute back to society)*
	Equal capability to be healthy
**Domain 3: Groupings of equalisation** *Avoiding negative discrimination based on personal factors or social determinates*	Discrimination
**Domain 4: Caveats to equalisation** *Prioritising care based on responsibility of the individual or the idea that all are entitled to a normal life span or quality of life*	Health behaviours of the individual *(behaviours that may directly affect an individuals’ health status)*
	Fair innings argument *(care should be distributed in terms of ensuring the opportunity for lifetime health)*
	Age *(priority should also be given to the young versus old who have had the opportunity to live)*
**Domain 5: Close enough** *Refers to an outcome that is not absolutely equal though is still considered to be good enough e.g. targeting ‘most people’ rather than ‘all people.’*	Close enough is good enough

Complete list of the five domains of equity and each sub-theme within each domain as described by Lane et al, 2017 (9). Information adapted from: Lane, Haylee, et al. "Equity in healthcare resource allocation decision making: a systematic review." Social science & medicine 175 (2017): 11-27

Two researchers (NG and MS) reviewed 10% of the triage policy tools independently. Any concerns during coding were noted and clarified between the two researchers. The remaining triage tools were then coded by one researcher (NG) and checked by another senior researcher upon completion (MS). This coding was then cross-checked by a clinician-researcher (KH) to ensure reliability and validity of coding. All authors involved in data analysis had a background in clinical physiotherapy and an understanding of using triage tools in hospitals.

Descriptive summative statistics were used to analyse and present the frequency of reported equity domains. The frequency of equity domains reported were categorised into standardised time frames for inpatient and outpatient settings to represent the proportionality within each category. The standardised timeframes for inpatient and outpatient settings used for analysis are included in Table 2.

**Table 2:** Standardised timeframes for triage tools in inpatient and outpatient settings used for analysis

Inpatient	Outpatient
0–8 h	0–3 days
8–24 h	3–7 days
24–48 h	7–14 days
48–72 h	15–30 days
72 + hours	31–90 days
not indicated/AHA^a^/when time^b^	90–365 days
not specified	not eligible for review
	not specified

Abbreviation: *AHA* Allied health assistant

^a^Indicated for allied health assistant to review patient

^b^Physiotherapy to review patient when they have time

The reporting of this manuscript adheres to the Standards for Reporting Qualitative Research checklist (Additional file 1).

## Results

A total of 13 prioritisation tools from 10 different organisations were collected and analysed. The deidentified information from the included triage policy tools and characteristics of the healthcare settings from which these tools were obtained are shown in Table 3. Public hospitals located in metropolitan regions comprised the majority of the sample and tools were primarily used in inpatient settings. Included tools contained both single-page risk matrices and multi-page documents used by physiotherapists or administration support, ranging from one page to 15 pages in length.

**Table 3:** Characteristics of organisations and triage tools

Characteristic	Frequency (%) of organisations *n* = 10
***Location of organisation with Australia***	
New South Wales	6 (60%)
Victoria	3 (30%)
Queensland	1 (10%)
***Metropolitan hospital***	9 (90%)
***Rural hospital***	1 (10%)
***Healthcare sector***	
Public	8 (80%)
Private	2 (20%)
***Setting of triage tool, n*** ** = ** ***13***	
Inpatient	8 (62%)
Outpatient	3 (23%)
Community	2 (15%)
***Time frame used by inpatient triage tools, n*** ** = ** ***39***	
0–8 h	11 (28%)
8–24 h	8 (21%)
24–48 h	6 (15%)
48–72 h	7 (18%)
72 + hours	1 (3%)
Not indicated / AHA / when time	6 (15%)
Not specified	
***Time frame used by outpatient/community triage tools, n*** ** = ** ***18***	
0–3 days	3 (17%)
3–7 days	2 (11%)
7–14 days	3 (17%)
15–30 days	4 (22%)
31–90 days	3 (17%)
90–365 days	1 (6%)
not eligible	1 (6%)
not specified	1 (6%)

*Abbreviation*: *AHA* Allied health assistant

**Fig. 1:**
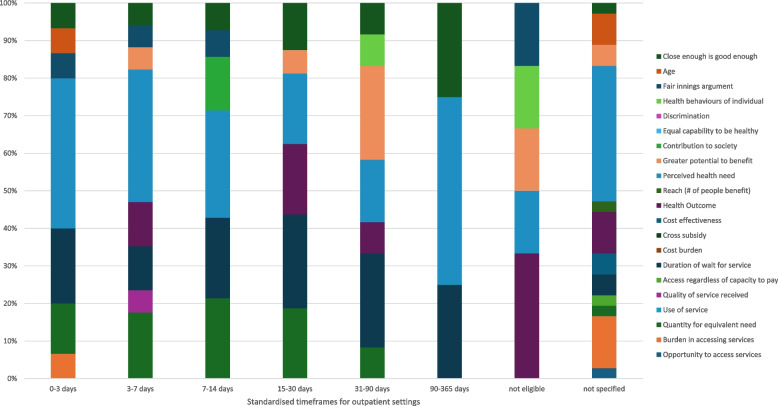
Representation of the frequency of references to each domain of equity in outpatient triage policy documents as a proportion within each standardised time frame

**Fig. 2:**
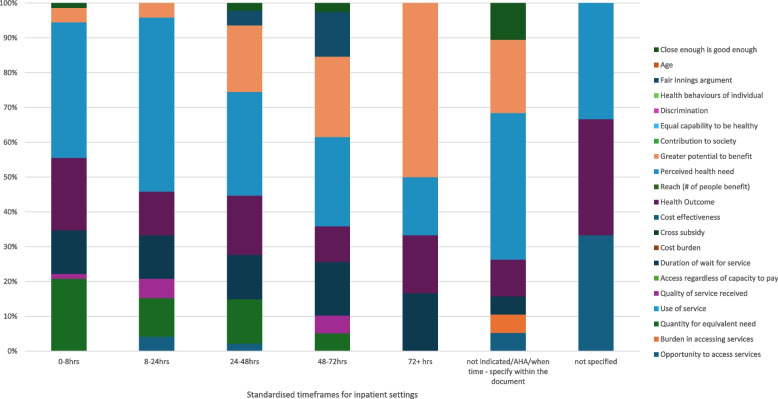
Representation of the frequency of references to each domain of equity in inpatient triage policy documents as a proportion within each standardised time frame

In total, four out of five themes of equity, including 16 sub-themes proposed by Lane et al. [9] (Fig. 1), were identified in the included tools. Example quotations relating to each equity sub-theme can be seen in Table 4.. The levels of prioritisation ranged from a maximum of seven different priority levels to a binary “eligible or not” criteria for physiotherapy. Across the 13 tools, themes of equity were mentioned 377 times (inpatient setting = 258 references, outpatient/community settings = 119 references). Figures 1 and 2 demonstrate the frequency of references to equity by each tool according to the standardised timeframes (Table 1.) and the proportionality within each category. Higher priority categories, particularly in inpatient settings, referenced equity themes more frequently with a lower number of references in lower priority categories (Fig. 1, Fig. 2). For example, there were 72 references to an equity theme for the ‘0–8 h’ (inpatient setting) category compared to 6 references for the 72 h or more category.

**Table 4:** Example text from triage policy documents representing each domain and sub-theme of equity

Equity Domain	Sub-theme	Quotation
**Domain 1: Point of equalisation in the health service supply/access/outcome chain**	1.1 Equal opportunity to access services	*“Blanket referrals to Physiotherapy and Occupational Therapy for all patients admitted to Rehabilitation”* [Organisation 6]
		*“Clarify how physiotherapy services are prioritised to provide the best access for patients”* [Organisation 4a]
	1.2 Equal burden in accessing services	*“If the patient lives closer to an alternative hospital, they will be contacted to be offered an interhospital transfer”* [Organisation 2]
		*“Suggestions of local physiotherapy providers or community services that can assist can be provided if suitable”* [Organisation 8]
		*“[Removed for anonymity] are unable to accept patient”* [Organisation 3b]
	1.3 Equal quantity for equivalent need	*“Receive a maximum of 8 sessions”* [Organisation 4c]
		*“The first treatment of patients on a surgical pathway requiring 2* × *per day treatment”* [Organisation 1]
		*“Day 1 and 2 post op invasive surgical including: abdominal, cardiothoracic, orthopaedic, spinal, vascular”* [Organisation 7]
	1.5 Equal quality of service received	*“Casting when the patient doesn’t have a suitable well fitting cast *in situ*”* [Organisation 9]
		*“Fracture clinic referrals post removal of cast if exercises not prescribed in fracture clinic”* [Organisation 3b]
	1.6 Equal access regardless of capacity to pay	*“If the patient has private insurance for physiotherapy they are encouraged to seek treatment in the private sector. If they so choose, they may still receive treatment at [name of] Hospital.”* [Organisation 2]
	1.7 Equalising duration of wait for service	*“To be seen within 30 min of referral [regardless of time of day]”* [Organisation 1]
		*“An appointment within 365 days is desirable”* [Organisation 2]
	1.10 Cost effectiveness	*“To identify patients who may be eligible for physiotherapy *via* other options such as a closer hospital, private physiotherapy (work cover, insurance cover, Enhanced Primary Care) or a Rehabilitation Unit.”* [Organisation 2]
	1.11 Equalising health outcome	*“Home based pulmonary or cardiac rehab for client with no recent change in their condition”* [Organisation 4c]
	1.12 Reach (no. of people benefit)	*“A number of outpatient groups/classes”* [Organisation 4b]
**Domain 2: Need or Potential to benefit**	2.1 Perceived health need	*“The specific objectives [of the triage document] are to:* *…provide guidelines for allied health staff regarding:* • *triage of patients referred to allied health to manage risks prioritisation for referrals based on patient need and resources…”* [Organisation 6]
		*“Patients requiring daily physiotherapy assessment and review to facilitate discharge”* [Organisation 3a]
		*“Clients who would not have functional deterioration without intervention"* [Organisation 3b]
		*“Allied Health intervention/assessment is required for a patient/client considered at high risk of harm”* [Organisation 6]
	2.2 Greater potential to benefit	*“Patients with acute increase work of breathing amenable to Physiotherapy”* [Organisation 3a]
		*“Patient/client with a recent acute event/exacerbation of chronic condition where evidence indicates that Allied Health intervention will result in a significantly improved client outcome and prevent adverse outcomes”* [Organisation 6]
		*“Patients classified ‘maintenance status’ and slow to progress patients”* [Organisation 1]
	2.3 Contribution to society	*“Pain which is stopping the patient living independently in the community”*
		“*Patient currently employed, unable to resume work”* [Organisation 4b]
**Domain 4: Caveats to equalisation**	4.1 Health behaviours of individual	*“Poor compliance with attendance, advice *etc.*”* [Organisation 4b]
		*“Falls caused by excessive ETOH (alcohol) use and illicit drug use which needs to be managed prior to physiotherapy”* [Organisation 5]
	4.2 Fair innings argument	*“Patients accepted to and awaiting rehab”* [Organisation 7]
		*“Patients that already have had intensive inpatient rehabilitation”* [Organisation 5]
		*“LBP (low back pain) patient who is mobilising independently with adequate pain relief and has been referred to outpatient physiotherapy.”* [Organisation 9]
	4.3 Age	*“Paediatric Outpatient Physiotherapy also requires a referral but there is no waitlist”* [Organisation 4b]
**Domain 5: Close enough**	5.1 Close enough is good enough	*“Time frames will be dependent on demand for services and resources available.”* [Organisation 2]
		*“seen when time.”* [Organisation 4a]
		*“to be seen in 30 min"* [Organisation 1]
		*“aim daily, minimum every 2nd/3rd daily.”* [Organisation 1]

*Abbreviations*: *LBP *Lower back pain

Three tools from rural healthcare settings (23%) were included in the sample with the remaining being from metropolitan areas (*n* = 10, 77%). In comparing outpatient triage tools, rural settings did not reference several domains that were included in metropolitan contexts including: ‘opportunity to access services’, ‘quality of service received’, ‘access regardless of capacity to pay’, ‘reach’, and ‘contribution to society’. Similarly, for inpatient tools, the following domains were not referenced in rural areas but were included in metropolitan areas; ‘quality of service received’, ‘duration of service wait’, and ‘fair innings argument’.

### Analysis of equity themes according to the five themes of equity

#### Theme 1: point of equalisation in the health service supply/access/outcome chain

Seven domains within the theme ‘point of equalisation in the health service supply/access/outcome chain’ were identified in the documents as key criteria for ensuring the equitable distribution of allied health services. Equal ‘opportunity to access services’ was identified in two organisations (*n* = 6 references) where blanket physiotherapy referrals would be applied in the subacute triage process unless the patient’s condition was explicitly considered “non-urgent.”

‘Equal burden in accessing services’ was referenced by four organisations (*n* = 7 references), mostly in relation to an individuals’ residential address. For example, an individuals’ proximity to an outpatient clinic would be used to determine where an individual would receive treatment. Alternatively, some outpatient clinics prioritised patients if they were unable to be seen by other services. ‘Quantity for equivalent need’ was also considered to determine the number of treatment services that individuals should receive. This application of equity was referenced on 43 occasions (10 organisations) in surgical pathways that specified particular presentations should be seen an equal amount. The implication being that all patients within the specified surgical pathway category should receive an equal quantity of physiotherapy on particular days.

‘Duration of wait for service’ was frequently referenced (*n *= 50 references, 11 organisations) across inpatient and outpatient settings. This sub-theme was evident in descriptions of various conditions all categorised under the same prioritisation level with a recommendation of when individuals should be seen. Time frames for patient review varied and depended on priority level e.g., high priority patients should be seen within 30 min of referral compared to the lowest priority, where it was acceptable to organise an appointment within 365 days of referral. Achieving equal ‘health outcomes’ or improvement in health states was considered in 12 organisations (52 references), often framed concurrently in the context of organisational or logistical goals. For example, patients were usually categorised according to health states with aims to prevent admission to intensive care units, reduce hospital length of stay, or facilitate discharge.

Sub-themes less frequently referenced included ‘equal access regardless of capacity to pay’ (*n* = 1 reference, 1 organisation), ‘cost effectiveness’ (*n* = 2 references, 2 organisations) and ‘reach’ (*n* = 1 reference, 1 organisation) which were identified in documents pertaining to outpatient or community settings.

#### Theme 2: need or potential to benefit

The most frequently referenced sub-theme was ‘perceived health need’, identified on 135 occasions (13 organisations). This theme was often central to the objective of prioritisation tools, denoted by concepts such as risk of deterioration or framing sentences with words like “required”. The theme of perceived need was frequently used in the inpatient setting as a stratified descriptor that correlated with different prioritisation levels, where resources would be allocated to those at higher risk of deterioration or could be used as a rationale for prioritisng patients.

Less prominent was the sub-theme ‘greater potential to benefit’, where 38 references were noted throughout the documents from 10 organisations through phrases such as “amenable to physiotherapy” or simply by the word “benefit”. Having the potential to benefit was often referenced in high priority patients or those considered “at high risk of harm”, where terminology would refer to an urgent perceived need for allied health intervention to reduce harm to the individual. Comparatively, lower priority level categories implied that there was either; a) existing evidence that reviewing non-urgent patients would result in additional benefit or b) little to no additional benefit, for example, patients prioritised as ‘maintenance status.’ The sub-theme ‘contribution to society’ was identified in one outpatient priority document, referencing community participation and return to work as a means for prioritising patient review.

#### Theme 4: caveats to equalisation

In outpatient and community settings, patient responsibility (*n* = 2 references, 2 organisations) was used to allocate resources where patients with “poor compliance with attendance, advice etc.” would be deprioritised. Where an individual’s health state was reduced due to risk-taking behaviours, such as in the case of falls caused by illicit drug use, patients may be deemed ineligible for some services until other behaviours were managed.

Triage tools from four organisations indicated that patients considered relatively low priority status encompass those who had been admitted to rehabilitation and were awaiting discharge from their acute inpatient phase. This classification aligned with the ‘Fair Innings Argument’ (*n* = 11 references, 6 organisations), suggesting that these patients have either received appropriate care for their current condition or have plans in place to do so, and care can therefore be distributed elsewhere. Similarly, community services would categorise patients as lower priority if they had already received suitable care in the inpatient setting, and inpatient services would deprioritise patients if they had outpatient referrals.

#### Theme 5: close enough is good enough

The theme of ‘close enough is good enough’ was identified on 13 occasions (6 organisations) in both inpatient and outpatient settings. The influence of time and/or resources was considered when prioritising patients, where high priority patients were allocated a specific timeframe in which to be reviewed. In contrast, timeframes for low priority patients were less specific, where allied health could “see when time.” These references implied that a health system could still be regarded as equitable, even when it allows for certain individuals to be in a less favourable health state or receive fewer resources than others.

## Discussion

This content analysis has identified the domains of equity incorporated into Australian hospital physiotherapy triage tools which influence the distribution of resources within healthcare organisations. Allocating resources based on perceived patient need and overall health outcome were the central guiding principles across both inpatient and outpatient settings. Additionally, the notions of providing an equal quantity of service relative to patient need and allocating services with the intent to reduce wait times emerged as significant considerations. There was limited explicit inclusion of several domains of equity including discrimination, equal capability to be healthy and other patient factors within the triage tools.

The design of triage tools can influence equity within health system structures and patient health outcomes when operationalised in different ways [8, 18]. Allied health professionals can both directly and indirectly affect health equity by ensuring equity-focussed care through parity in access and adequate consideration of the social determinants of health [19]. When the principles of equity are not adequately considered, healthcare inequalities can be exacerbated [19]. In this analysis, different domains of equity were operationalised in each triage tool, either implicitly or explicitly, with the prioritisation of patients with the greatest 'need' based on their health state emerging as a common justification for resource distribution. The rationale behind this theme in triage may be grounded in how a health ‘need’ is considered evidence-based and whether there is clear evidence that a service will directly impact the health outcomes of the target population [10]. For example, multiple tools referred to a patient at risk of life-threatening deterioration, and that these patients would be prioritised for treatment where there is evidence that a physiotherapy intervention can improve health outcomes, over a patient at low risk of deterioration, and review would result in ‘functional gains only’. However, a critical consideration is the subsequent effects of using different equity domains to guide resource allocation. Studies examining the distribution of health services, including allied health, have shown that whilst higher priority patients according to need have shorter wait times, lower priority patients subsequently have longer than intended wait times [20, 21]. Further, clinical resources can sometimes be diverted from outpatient services to inpatient services to prioritise higher acuity patients [22]. It is crucial when designing triage tools to carefully consider the stages within the healthcare supply, access or outcomes chain that could be equalised (i.e. points of equalisation) to avoid worsening disparities in healthcare delivery [23].

Determining the point of equalisation for achieving health service supply, access or outcome equity can be a challenging task and requires a balancing act between the principles of horizontal equity (people with equal needs should be treated the same) and vertical equity (people with greater clinical needs should have more intervention than those with lesser needs) [24, 25]. Studies have highlighted the complexity of this task, where stakeholders are able to discuss potential domains of equity but have struggled to select a single point of equalisation to guide resource allocation decisions or reach a consensus on which domain should take precedence [10, 22]. Policies may be dependent on the context in which the policy to guide resource distribution is intended. Historically, triage policies have tended to be guided by the principle of saving the most lives and directing resources to those most urgently in need [9, 26]. However, the onset of the COVID-19 pandemic has precipitated scrutiny due to evidence that health disparities have been exacerbated, prompting calls for a reevaluation of triage policies to incorporate both principles that save the most lives and mitigate health inequities [27]. Attempting to tackle these disparities is not new. For example, when the NHS implemented a new resource allocation policy that prioritised deprived areas, they used the underlying rationale that additional healthcare expenditure would translate to improved population health outcomes. As a result, the increase in resources allocated to each area between 2001 and 2011 was associated with a reduction in mortality amenable to reallocated healthcare services [28].

Another contextual consideration may be the differences between rural and metropolitan areas, where the use of various points of equalisation may impact populations differently. Interestingly in our study, outpatient triage documents from rural settings did not reference themes such as service utilisation, societal contribution, or access opportunities as points of equalisation, unlike metropolitan organisations. Instead, rural settings emphasised resource allocation based on two overarching ideas. Firstly, that healthcare should be distributed in terms of opportunity for lifetime health (‘fair innings argument’) and secondly, that individual health behaviours which may directly affect their health status should be considered when prioritising care [9]. This may reflect the needs and health risks of the population groups that live in rural areas where people living in rural or remote areas face unique challenges, such as poorer access to health services, leading to poorer health outcomes[29]. Additionally, there may be differing considerations of equity principles between rural and metropolitan contexts. How these differences may impact different populations could be further explored.

In this study, neither inpatient nor outpatient triage documents referenced the theme ‘groupings of equalisation.’ Outside the context of allied health, the avoidance of negative discrimination based on personal factors, such as social determinants, is often a central consideration in addressing equity. For example, in emergency department settings, inequities in the provision of health care and health outcomes such as the assignment of lower triage acuity scores or increased risk of readmissions to emergency departments, have been reported in patients who use a primary language for care other than English [30, 31]. To address disparities stemming from race, ethnicity, and language barriers, emergency departments have focused efforts by targeting implicit bias, structural racism, and language barriers [30–32]. Similarly, in the context of COVID-19 and vaccine equity, considerations of income disparities among populations in various countries have been acknowledged, with a core focus on ensuring equal opportunity to access vaccines grounded in the principle that everyone should be able to attain the highest standard of health [33–36]*.* This prompts consideration of whether all policy documents should be made publicly available, in formats that are appropriate for diverse populations, so that health consumers can be more informed about how care is allocated, and the “substance of deliberation" involved in making decisions under resource constraints [37].

There were some limitations to this study. A relatively small sample of documents from one country, within predominantly metropolitan areas, were analysed which may limit the generalisability of findings to other hospitals outside of the Australian context. We also focussed findings on physiotherapy services which may not be representative of all allied healthcare and certain domains of equity may have been addressed in other healthcare services beyond physiotherapy and in organisations not recruited to the study. Whilst triage tools are a core way of allocating resources, solely focusing on formal, written documents may potentially overlook informal methods of allocating resources, such as leveraging personal connections with other healthcare professionals. Including a variety of healthcare services from a larger number of organisations is important for future studies to gain a complete picture of the how the principles of equity have been applied within healthcare settings. More detailed analysis of grey literature/publicly advertised values of government health departments may also be useful to demonstrate the difference between intended and implemented definitions of equity.

## Conclusions

This study provides a snapshot of the different domains of equity that have been operationalised in physiotherapy triage tools within Australian hospitals, to explore how physiotherapists prioritise patient care across inpatient and outpatient settings. The analysis demonstrates that several domains of equity are considered during the triage process to guide resource allocation decisions, namely the perceived health need of a patient and their potential to benefit. Equal quantity of service (relative to need), health outcomes (albeit institutional rather than clinical health outcomes), and wait times were also found to be important to the process of prioritisation. There was limited consideration of several domains of equity including discrimination, which is a focal consideration of how equity considerations are addressed outside the context of triage. While our analysis refrains from advocating for a specific equity definition in triage settings, or what should be used, it seeks to outline which definition has been applied in the triage of hospital physiotherapy services. Policymakers and service providers can leverage the insights gleaned from this study to review the application and operationalisation of equity principles within their healthcare systems through mechanisms such as patient triage tools.

### Supplementary Information

Additional file 1. Standards for Reporting Qualitative Research (SRQR) checklist: Populated checklist of ‘Standards for Reporting Qualitative Research’ reporting guidelines indicating how the manuscript adheres to the relevant guidelines

## Data Availability

The datasets used and/or analysed during the current study are available from the corresponding author on reasonable request.
